# Supplementary material for: Machine-learning model led design to experimentally test species thermal limits: The case of kissing bugs (Triatominae)
Source: PLoS Negl Trop Dis. 2021 Mar 8;15(3):e0008822. doi: 10.1371/journal.pntd.0008822 (PMC7971882; doi:10.1371/journal.pntd.0008822)
Supplement: S1 Fig — (DOCX) [file pntd.0008822.s001.docx]

**Supplementary information S1 Fig**

Machine-learning model led design to experimentally test species thermal limits

J. E. Rabinovich, A. Alvarez Costa, I. Muñoz, P. E. Schilman & N. M. Fountain-Jones

**Additional figures**

**Interaction between exposure time and species.**


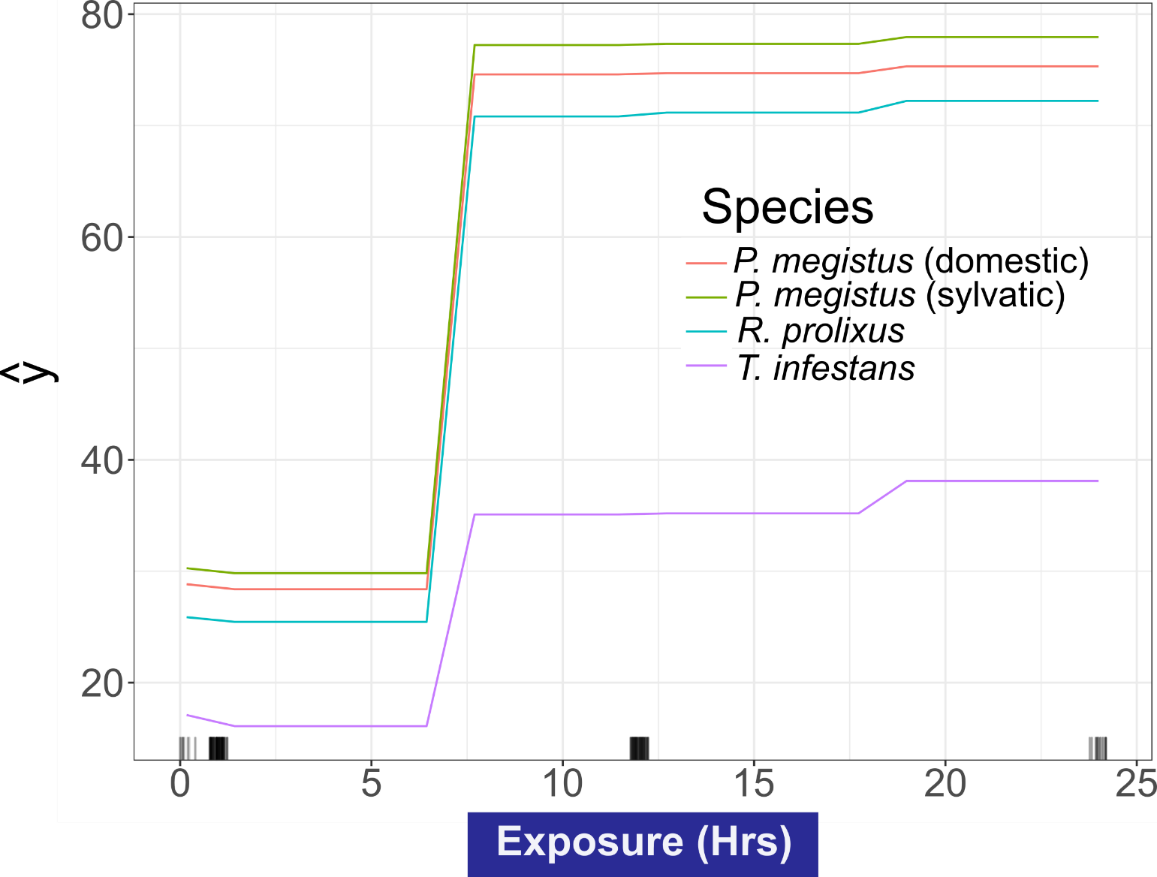


Fig. A. 2D partial dependency plot from the best performing machine learning model (GBM), showing the interaction between exposure time and species. Here $\hat{y}$ is the % estimated mortality.

**Survival curves (*l_x_*, as a function of *x*, number of days after the heat treatment).**

Fig. B. Survival curves (*l_x_*, as a function of *x*, number of days after the heat treatment) from the survival curves predicted by the Cox analysis. The ribbons are the 95% confidence intervals.
